# In vitro and in vivo anti-diabetic and anti-oxidant activities of methanolic leaf extracts of Ocimum canum

**DOI:** 10.22088/cjim.10.2.162

**Published:** 2019

**Authors:** Chimaobi J. Ononamadu, Adamu J. Alhassan, Abdullahi A. Imam, Aminu Ibrahim, Godwin O. Ihegboro, Alowonle T. Owolarafe, Mohammed S. Sule

**Affiliations:** 1 Department of Biochemistry and Forensic Science, Nigeria Police Academy, Wudil, Kano State, Nigeria; 2 Department of Biochemistry, Bayero University, Kano State, Nigeria

**Keywords:** Diabetes mellitus, Oxidative stress, Ocimum canum, insulin

## Abstract

**Background:**

Diabetes is a metabolic disorder with a highly complex, multifaceted and intricate etiologies and thus may require management options that proffers multimodal mechanism of action. This present study evaluated the antidiabetic and antioxidant potential of the methanolic extract/fractions of leaves of *Ocimum canum.*

**Methods:**

The antidiabetic potential was evaluated and using STZ-induced diabetic Wistar rat model (*in vivo*) and inhibition of α-amylase and α-glucosidase activity (*in vitro*). Antioxidant activity was assessed *in vitro* by free radical scavenging and reducing power assays and *in vivo* via monitoring SOD and CAT activities; GSH and MDA levels.

**Results:**

The total phenolic content (221.0±3.0 mg catechol/g of sample) and tannins (146.0±4.0 mg tannic acid/g of sample) of the crude extract; and flavonoid of the aqueous-methanol fraction (216.0.0±1.0 mg of rutin/g of sample) were found to be significantly higher relative to others. The crude extract and the aqueous-methanol fraction exhibited a significantly (p<0.05) higher percentage reduction in fasting blood glucose and a concomitant increase in serum insulin level relative to the diabetic control group. The highest radical scavenging activity and reducing power were observed in the aqueous-methanol fraction. The aqueous-methanol solvent fraction also significantly reversed the alterations in oxidative stress markers occasioned by the diabetic condition.

**Conclusion:**

In conclusion, the result of the present study has demonstrated evidently that extracts of *Ocimum canum* leaves ameliorates hyperglycemia and the associated oxidative stress in STZ-induced rats.

Diabetes mellitus is a chronic metabolic disorder characterized by hyperglycemia, hypertriglyceridemia and hypercholesterolemia, which results from defects in insulin secretion, its actions or both (1). The disorder is associated with an imbalance of the glycemic index and glucose intolerance, which increases the risk of individuals progressing to type 2 diabetes mellitus. Complications from diabetes, such as coronary artery and peripheral vascular diseases, stroke, diabetic neuropathy, risk of ulcers and amputations, renal failure, sexual dysfunction, and blindness accounts for the increasing disability, reduced life expectancy, and enormous health costs associated with this condition for virtually every society (2). The disease has attained great significance in the sub-Saharan region, with Nigeria being the most affected. Put in numbers, there are about 387 million people with diabetes globally, of which 22 million reside in sub-Saharan Africa (3). It is becoming the third killer of mankind after cancer and cardiovascular disease due to its high prevalence, morbidity and mortality (4). 

The disease is a disorder with a complex and multifaceted etiology. The oxidative stress of hyperglycemia has extensively been implicated to be a central mechanism. For development, progression and complications of the condition, thus this necessitates the development of antidiabetic drugs with multimodal mechanism of action. Most of the antidiabetic drugs currently available act via one known mechanism and may also present with some undesirable effect when used. As alternatives, herbal remedy may provide a comparative advantage by reason of the presence or possession of diverse bioactive secondary metabolites which could exert their effect via different mechanisms (2, 4). 

Plants have been the basis of traditional medicine systems around the world for thousands of years. Even in modern-medicine systems, ethnobotanical treatments continue to play an important role in healthcare. About 80% of the populations in developing countries rely on traditional medicine for their healthcare (2). In Nigeria, *Ocimum* genus species form a significant part of the folk medicine system for the management of diabetes mellitus. However, this practice still suffers from lack of scientific validation of their efficacy, dosage, safety, and mechanisms of action. Furthermore, they represent a potential major source of lead compounds for treatment of diabetes mellitus.

*Ocimum canum* is common specie in the genus of *Ocimum*. It is a natural growing herb, but is cultivated in some climes. It is characterized by a pungent aromatic flavor and commonly cultivated for culinary and medicinal purposes. It is used in preparing different local dishes as a spice as well as vegetable. It is a source of fragrance and aroma compounds (5, 6).

The plant is used in traditional medicines to treat conjunctivitis, malaria and headache, high blood sugar and other ailments (7, 8).

 Despite the use in management of metabolic conditions such as diabetes, there is still dearth of extensive scientific report on the antioxidant and antidiabetic activity of the leaves of this plant. 

Previously, Nyarko et al., and Dash et al. reported the hypoglycemic potentials of this plant, however, despite these studies, there are still some knowledge gaps that requires further work (9-11). This present study intends to screen the methanolic extract of the leaves of Ocimum canum for antioxidant and hypoglycemic activity as a first phase of characterization of this plant.

## Methods

**Experimental Animals: **This research was carried out using fifty seven (57) Wistar rats of 150-180g weight range. The animals were obtained from the Department of Biological Sciences, Bayero University Kano State, housed and maintained at normal room temperature. They were placed on standard commercial feeds and clean water *ad libitum *and allowed to acclimatize for 2 weeks before the work proceeded*.*

**Plant materials:** *Ocimum canum *leaves were obtained from Yankura Market in Sabon gari, Kano, and was identified and authenticated (voucher No: BUKHAN0306) by a taxonomist in the Department of Biological Science, Bayero University Kano State. 

**Chemicals: **All chemicals and kits used were of analytical grade

**Preparation of plant extracts: **The extract and solvent fractions were prepared as described by Teke *et al. *with little modifications (12). The plant leaves were shade dried and coarsely powdered. A mass of 500 g of the ground leaves was macerated in 2.5L of methanol for 72 hours. It was then filtered and the solvent was evaporated under reduced pressure in a rotary evaporator at 50°C to afford 32g of methanol extract of *Ocimum canum*. The extract was labeled and preserved for further use. A mass of 20.14 g of this extract was pre-dissolved in 100 cm^3^ of methanol and then partitioned into n-hexane (350 cm^3 ^x3) and the upper (n-hexane) phase was collected and concentrated as n-hexane solvent fraction. The residual methanol phase was collected, concentrated and then re-dissolved in 100cm^3^ of aqueous-methanol (55:45 v/v) and portioned into ethyl acetate (350 ml x3). The resulting upper (ethyl acetate) phase was collected and concentrated as the ethyl acetate solvent fraction while the lower (aqueous-methanol) phase was collected and concentrated as the aqueous-methanol solvent fraction. All extract and fractions were labeled and preserved for further use.

**Phytochemical analysis: **The phytochemical analysis of the extracts and fractions was carried out using the methods described by Sofowora (13**)**, Harbourne (14) and Saeed *et al.* (15).

**Alkaloids: ** A volume of 1cmᶟ of 1% HCl was added to 3 cmᶟ of the extracts in a test tube. The mixture was heated for 20 minutes, cooled and then filtered. The filtrate was used as follows.

- Two drops of Mayers reagent were added to 1cmᶟ of the extracts. A creamy precipitate indicated the presence of alkaloids in the extracts.
- Two drops of Wagner’s reagent were added to 1cmᶟ of the extracts. A reddish brown precipitate indicated the presence of alkaloids

**Tannins: ** A volume of 1cmᶟ of freshly prepared 10%w/v KOH was added to 1cmᶟ of the extracts. A dirty white precipitate indicated the presence of tannins. 

**Phenolics: **Two drops of 5% FeCl₃ were added to 1cmᶟ of the extracts in a test tube. A greenish precipitate indicated the presence of phenolics. 

**Glycosides: **A aliquot of 10 cmᶟ of 50% H₂SO₄ was added to 1cmᶟ of the extracts, the mixture was heated in boiling water for 15 minutes. Then, 10 cmᶟ of Fehling’s solution was added and the mixture boiled. A brick red precipitate indicated the presence of glycosides.

**Saponins:** Frothing test: A volume of 2 cmᶟ of the extract in a test tube was vigorously shaken for 2 minutes. Frothing indicated the presence of saponins.

- Emulsion test: Five drops of olive oil were added to 3 cmᶟ of the extract in a test tube and vigorously shaken. A stable emulsion formed indicated the presence of saponins.

**Flavonoids: **An aliquot of 1cmᶟ of 10% NaOH was added to 3cmᶟ of the extracts. A yellow colouration indicated the presence of flavonoids.

**Steroids: **Salkowsti test: Five drops of concentrated H₂SO₄ were added to 1cmᶟ of the extracts. A red colouration indicated the presence of steroids

**Phlobatannins:** An aliquot of 1cmᶟ of the extracts was added to 1%v/v HCl. A red precipitate indicated the presence of phlobatannins.

**Triterpenes: **Five drops of acetic anhydride were added to 1cmᶟ of the extracts. A drop of concentrated H₂SO₄ was then added and the mixture was steamed for 1 hour and neutralized with NaOH followed by the addition of chloroform. A blue green color indicated the presence of triterpenes.

**Carotenoids: **A gram of each sample was extracted with 10 ml of chloroform in a test tube with vigorous shaking. The resulting mixture was filtered and 85% sulphuric acid was added. A blue color at the interface showed the presence of carotenoids.

**Estimation of total phenolic content: **The total phenolic content was determined spectrophotometrically according to the method described by Saeed *et al. *(15) with slight modifications. To a 10 cm^3^ test tube containing 0.1cm^3^ of extracts (in triplicates), 5.0 cm^3^ of distilled water and 0.5 cm^3^ of (10% v/v) Folin-Ciocalteu’s phenol reagent prepared in water were added sequentially and shaken. After 5 minutes, 0.5 cm^3^ of 2% Na_2_CO_3_ solution was added and mixed thoroughly. The mixture was kept in the dark for 30 min at room temperature, after which the absorbance was read against a blank (all reagents with methanol substituting the extract) at 765 nm. The standard curve for total phenolics was made using catechol standard solution (0 to 100 mg/l) following the same procedure as earlier described for the extracts. The total phenolics were determined from the standard curve and expressed as milligrams of catechol equivalent per g of dried extracts (crude extracts or fractions)

**Estimation of total flavonoid content: **Total flavonoid content was determined following a method by *Saeed et al.* (12) with slight modifications. To a 10 cm^3^ test tube containing 0.3 cm^3^of extracts (in triplicates), 3.4 cm^3^ of 30% methanol, 0.15 cm^3^ of NaNO_2_ (0.5 M) and 0.15 cm^3^ of aluminium chloride (AlCl_3_.6H_2_O (0.3 M)) were added sequentially. After 5 min, 1 cm^3^ of NaOH (1 M) was added. The solution was shaken to mix well and the absorbance of the resulting solutions was measured against the reagent blank (all reagents with methanol substituting extract) at 506 nm. The standard curve for total flavonoids was made using a standard solution of rutin (0 to 100 mg/l) following the same procedure as earlier described for the extracts. The total flavonoids were determined from the standard curve and expressed as milligrams of rutin equivalentper g of extracts (crude extracts or fractions).

**Determination of tannin content: **The tannins were determined by the Folin - Ciocalteu method according to Saeed *et al.* (15) with slight modifications. To a 10 cm^3^ test tube containing 0.1 ml of the sample extract (in triplicates), 7.5 cm^3^ of distilled water and 0.5 cm^3 ^of Folin-Ciocalteu phenol reagent was added and shaken, after 5 mins, 1cm^3^ of 2 % Na_2_CO_3_ solution was added and mixed thoroughly. The mixture was kept in the dark at room temperature for 30 mins to develop color. The absorbance for the test and standard solutions was measured against a blank (all reagents with methanol substituting extract) at 725 nm. The standard curve for tannin was made using tannic acid standard solution (0 to 100 mg/l) following the same procedure as earlier described for the extracts. The tannin content was expressed as milligrams of (tannic acid equivalence) TAE/g of dried crude extract or fractions. 

**Determination of β-carotene and lycopene content: **B-carotene and lycopene were determined simultaneously according to the method of Nagata and Yamashita (16). A sample of extract weighing 0.1g was extracted with 4 cm^3^ of acetone-hexane (4:6 by volume) at 37^o^C for 10 min in triplicates. 

The resulting solutions were centrifuged and the absorbance of the supernatant taken at following wavelengths: 663 nm, 645 nm, 505 nm and 453 nm. The β-carotene and lycopene content was determined per 100cm^3 ^by the following equations and subsequently expressed per gram of sample:

Lycopene (mg/100 cm^3^) = -0.0458A_663 _+0.204A_645_ +0.372A_505_-0.0806A_453_

B-carotene (mg/100 cm^3^) = 0.216A_663 _- 1.22A_645_ - 0.304A_505_-0.452A_453_

Where A_663_, A_645_, A_505_ and A_453 are absorbance _at 663, 645, 505, and 452 nm

**α-Amylase and α-glucosidase inhibition assay: **The effect of the plant crude extract and solvent fractions on α-amylase and α-glucosidase activity was determined according to the method described by Kazeem *et al. *(17) with modifications. 

***α-amylase: ***To 250ul of each extract concentration in a test tube (0-360 ug/ml), the following were added sequentially: buffered α-amylase (250µL, 0.05 mg/ml), starch (250µL, 1%), the reaction mixture was incubated for 10 min at 25^o^C. DNSA (500µl) was added subsequently and then boiled for 5 mins. It was then cooled and diluted with 5 ml of dH_2_O. The control was prepared in the same manner as the test samples with distilled water replacing the extract. The absorbance of each test tube content was taken at 540 nm and the percentage inhibition was calculated as follows;

**% Inhibition=** $\frac{\mathrm{Ac}-\mathrm{At}}{\mathrm{Ac}}\mathrm{x 100}$ where A_c_ and A_t_ are the absorbance of the control and tests, respectively.

The concentration of the extracts resulting in 50% inhibition of the enzyme activity (IC_50_) was determined graphically.

**α-glucosidase : **To 50ul of each extract concentration in a test tube (0-40ug/ml) the following were added sequentially: buffered α-glucosidase (100µL, 1.0U/ ml) and incubated at 37^o^C for 10minutes, then pNPG (50µl, 3.0 mM) and incubated at 37^o^C, for 20 min, then Na_2_CO_3 _ (5%w/v), cooled to 25^o^C and lastly 5 ml H_2_O was added and vortexed. The absorbance of the yellow p-nitrophenol from the different test tubes will be taken at 405 nm and the percentage inhibition was calculated as follows;

**% Inhibition=** $\frac{\mathrm{Ac}-\mathrm{At}}{\mathrm{Ac}}\mathrm{x 100}$ where A_c_ and A_t_ are the absorbance of control and tests respectively

The concentration of the extracts resulting in 50% inhibition of the enzyme activity (IC_50_) will be determined graphically

**Acute toxicity (LD**_50_ **) study: **The acute toxicity (LD_50_) test of the methanol extract of leaves of *Ocimum canum* was determined using Lorke’s method (18). The crude methanol extracts were prepared in DMSO (10%v/v). The test was carried out in two phases. The first phase involved the use of nine (9). Wistar rats randomly distributed into three groups. The groups were administered 10, 100 and 1000 mg/kg body weight of the extract orally. The animals were monitored for 24 hours for gross behavior and mortality. The second phase involved three (3). Wistar rats. Three different doses of 1600, 2900 and 5000 mg/kg body weight of the extract were administered to each rat. The animals were monitored for 24 hours for mortality. The LD_50_ was calculated as the geometric mean of the maximum dose that caused 0% death and the minimum dose that caused 100% death.

**Induction of Diabetes: **Experimental diabetes was induced as described by Juarez-Rojop *et al. *(19) with modifications. Following overnight fasting, a single intraperitoneal injection of streptozotocin (60 mg/kgbw) dissolved in sterile water for injection was administered. This was followed by an oral administration of 5w/v% glucose solution two hours after induction. The control animals received the sterile water as placebo. The animals were checked for successful induction of diabetes after 48 hours. Animals with blood glucose above 300mg/dl were classified as diabetic. 

**Experimental Design: **The animals (45 in number) were weighed and randomly assigned to groups (n=5) of treatments as follows;

Grp 1 (NDBC): Normal rats administered with DMSO+ food and water 

Grp 2 (DBC): Diabetes – induced rats administered DMSO+food and water 

Grp 3 (CROC1): Diabetes – induced rats administered with 100 mg/kgbw of OC +food + H_2_O

Grp 4 (CROC2): Diabetes – induced rats administered with 200 mg/kgbw of OC +food + H_2_O 

Grp 5 (CROC3): Diabetes – induced rats administered with 300 mg/kgbw of OC +food + H_2_O

Grp 6 (HXOC): Diabetes – induced rats + hexane fraction of OC (100 mg/kgbw) +food + H_2_O

Grp 7 (ETOC): Diabetes – induced rats + ethylacetate fraction of OC (100 mg/kgbw) +food + H_2_O

Grp 8 (HMOC): Diabetes – induced rats + aqueous-methanol fraction of OC (100 mg/kgbw) +food + H_2_O

Grp 9 (GLMD): Diabetes –induced rats + 5 mg/kg bw of glibenclimide (standard drug) + food + H_2_O

**OC= Ocimum canum, DMSO: Dimethylsulfoxide**

**Determination of blood glucose and collection of blood and organ samples: **The treatments were administered daily for 28 and 15 days with the crude extract and the solvent fractions respectively. Blood glucose was determined on a daily basis using accucheck glucometer, blood samples were collected via a slight incision on the lateral tail vein using a scalpel blade. The measurements were taken in duplicates to ensure consistency in the glucometer readings. After the last day of treatment, the rats were sacrificed under light anesthesia following a 12-hour fast (19), blood samples were collected, centrifuged and the serum stored for biochemical assays. The pancreas and liver were excised immediately, washed with chilled isotonic saline and stored in 10% formal saline and at -4^o^C in phosphate buffered saline (1.0X) respectively for further analysis.

**Determination of serum insulin: **Serum insulin was determined using Accu Bind ELISA Microwell Insulin Test System (product code: 2425-300) according to the manufacturer’s manual.

**Histopathological assessment: **Histopathological examinations were carried out on the pancreas of the rats. They were fixed in 10% formalin, dehydrated in graded ethanol concentrations (50-100%), cleared in toluene and embedded in paraffin. Sections (4-6 μM thick) were prepared and then stained with Hematoxylin and Eosin (H-E) dye for photomicroscopic observation under light microscope at high power magnifications (x 400 objectives). The stained section was observed with Leica DMTSO microscope and photographed with Leica ICC50 HD camera (20).

**Determination of** ***in vitro*** **antioxidant activity of the extract: **The *in vitro *antioxidant activity of the methanol crude extract /solvent fractions of *Ocimum canum* were determined according to the methods described in Saeed *et al.* (15).

**DPPH assay (2, 2-diphenyl-1-picrylhydrazyl): **The free radical scavenging activity of the extracts was determined *in vitro* by 2,2′-diphenyl-1-picrylhydrazyl (DPPH) method. Briefly, DPPH stock solution (0.1mM) was prepared by dissolving 4 mg of DPPH in 100 ml methanol and stored at 20°C until required. The working solution was obtained by diluting the DPPH solution with methanol to attain an absorbance of about 1.2±0.09 at 517 nm using the spectrophotometer. A 3 cm^3^ aliquot of this solution was mixed with 100μl of the various concentrations (0-100 μg/cm^3^). The reaction mixture was shaken well and incubated in the dark for 30 min at room temperature. The absorbance was taken at 517 nm. The control was prepared as described above with methanol substituted for the sample. The % scavenging activity was determined by the following equation:

DPPH scavenging activity (%) = [(control absorbance−sample absorbance)/ (control absorbance)]×100

**Phosphomolybdate assay (total antioxidant capacity): **The total antioxidant capacity was evaluated by the reduction of phosphomolybdate (VI) to phosphomolybdate (V) blue complex (15). Briefly, an aliquot of 0.1cm^3^of sample solution was mixed with 1cm^3^of reagent solution (0.6M sulphuric acid, 28 mM sodium phosphate and 4 mM ammonium molybdate). The tubes were capped and incubated in a water bath at 95°C for 90 min. After the samples had cooled to room temperature, the absorbance of the mixture was measured at 765 nm against a blank. Ascorbic acid was used as standard. The antioxidant capacity was estimated using the following formula:

Total antioxidant capacity (%) = [(control absorbance – sample absorbance)/(control absorbance)]×100 

**Superoxide ion scavenging assay: **The superoxide scavenging activity of the extract was determined according to the method described in Saeed *et al*.’s study (15). Briefly, a 1cm^3^aliquot of different concentrations (0-150μg/cm^3^) of the extract was mixed with 0.5 ml of phosphate buffer (50 mM, pH 7.6), 0.3 cm^3 ^riboflavin (50 mM), 0.25 ml PMS (20 mM), and 0.1 ml NBT (0.5 mM) sequentially. The reaction was initiated by illuminating the reaction mixture using a fluorescent lamp. After 20 min of incubation, the absorbance was measured at 560 nm. Ascorbic acid was used as standard. The scavenging ability of the plant extract was determined by the following equation:

 Scavenging activity (%)=(1−(absorbance of sample/absorbance of control)×100

**Hydroxyl radical scavenging assay: **The superoxide scavenging activity of the extract was determined according to the method described in Saeed *et al*. study (15). Briefly, the reaction mixture contained; 500 μl of 2-deoxyribose (2.8 mM) in phosphate buffer (50 mM, pH 7.4), 200 μl of premixed ferric chloride (100 mM) and EDTA (100 mM) solution (1:1; v/v), 100 μl of H_2_O_2_ (200 mM) with 100μl graded concentrations (0-250μg/ml) of the extracts or without the extract for control. The reaction was triggered by adding 10μl of 300mM ascorbate and incubated for 1hour at 37°C. A 0.5cm^3^ aliquot of the reaction mixture was taken and added to 1 cm^3 ^of TCA (2.8% w/v aqueous solution), then 1cm^3^ of 1%w/v aqueous TBA was added to the reaction mixture. The mixture was heated for 15 min on a boiling water bath (100^o^C). The mixture was cooled and the absorbance at 532 nm was taken against a blank (the same solution but without the test solution). The hydroxyl scavenging activity of the extracts was calculated as follows:

Scavenging activity (%) = (1−absorbance of sample/absorbance of control) ×100

**Reducing Power assay: **The reducing power was based on Fe (III) to Fe (II) reductive transformation in the presence of the test samples that can be monitored by measuring the formation of Perl’s Prussian blue at 700 nm as described in (15). Briefly, various concentrations (0-200μg/ml) of the extract/fractions (2cm^3^) were mixed with 2cm^3^ of phosphate buffer (0.2 M, pH 6.6) and 2 cm^3^ of potassium ferricyanide (10 mg/cm^3^). The mixture was incubated at 50°C for 20 min followed by the addition of 2 cm^3^ of trichloroacetic acid (100 mg/l). The mixture was centrifuged at 3000 rpm for 10 min to collect the supernatant of the solution. A volume of 2 cm^3^ from each of the mixture earlier mentioned was mixed with 2cm^3^of distilled water and 0.8 cm^3^ of 0.1% (w/v) fresh ferric chloride. After 10-min reaction, the absorbance was measured at 700 nm. The higher absorbance of the reaction mixture indicates a higher reducing power. A standard curve of ferric sulphate was used to quantify the ferric reductive potential of the extract/fractions. IC_50_ for reducing power assay was determined as the concentration of extract / solvent fraction that gave a reduction equivalence of 50mM Fe^2+^

**Determination of** ***in vivo*** **antioxidant activity:**

**Tissue Preparation: **Tissue homogenate (10%) was prepared by homogenizing 1g of frozen tissue in 10 ml of 0.1M phosphate buffer at a pH of 7.4. The homogenate was centrifuged for 10 mins at 2500rpm/min. The supernatant was collected for the in vivo antioxidant assays.

**Reduced Glutathione and Glutathione Peroxidase (GSH-PX): **Reduced glutathione and glutathione peroxidase were determined according to the method described in the Elabscience GSH and GSH-Px assay kit (catalog No: BC0051) manufacturers manual.

**Superoxide Dismutase Activity (SOD): **SOD was determined according to the method described by Gavali *et al,* (21) with slight modification. This method is based on the competitive inhibition of the autoxidation of pyrogallol under alkaline medium by SOD. The degree of inhibition is proportioned to the SOD activity. Briefly, to an aliquot of 0.9 cm^3^ of phosphate buffered EDTA (1mM) solution, 0.05 cm^3^ of the homogenate was added in a test tube, and shaken to mix. The reaction was started by adding 0.05cm^3^ of pyrogallol solution (20 mM). The absorbance was read at 420mm exactly after 1 min and 30 secs. For the control, the procedure was repeated, but 0.1ml of buffer was added in place of the homogenate. The SOD activity was calculated as follows:

Inhibition ratio= Abs control - Abs sample x 100

 Abs sample

SOD activity (U/mg tissue/ml) = SOD inhibition ratio /50 x Conc of sample (mg/ml)

One unit of SOD is defined as the amount of the enzyme required to cause 50% inhibition of pyrogallol auto oxidation under the assay condition above

**Catalase Activity: **Catalase activity was determined according to the method described by Prabhakar *et al. *(22). The method is based on the rate of decomposition of H_2_O_2_ (hydrogen peroxide) by catalase which can be monitored at 230-240 mm spectrophotometrically. Briefly, to 2.95 cm^3^ of H_2_0_2_ solution (0.1%v/v), 50µl of the homogenate was added. The decrease in absorbance was monitored at 230 nm for one minute. The catalase activity was calculated and expressed as mmol of H_2_O_2_ decomposed per minute per milligram of tissue as shown below:

$\mathrm{Catalase}(U/\mathrm{mg tissue}/\mathrm{ml})=\frac{\mathrm{\Delta Abs}/\mathrm{minute}\times \mathrm{1000}}{43.6\times \mathrm{conc of tissue in sample}}$

**Lipid Peroxidation: **MDA was determined according to the method described by Prabhakar *et al.* (22) with slight modification. The method is based on MDA (the product of lipid Peroxidation) forming a complex that absorbs maximally at 532 nm with thiobatbituric acid (TBA). Briefly,** a**n aliquot of 0.2 cm^3^ of the tissue homogenate was mixed with, 2 cm^3^ of thiobarbituric acid (TBA (0.375%)) -trichloroacetic acid (TCA (15%)) reagent. The volume was made up to 3 cm^3^ with distilled water and then it was boiled in water bath at 95^o^C for 20 mins. The solution was then cooled and the reaction product TBA – MDA complex was extracted by adding 3 cm^3^ of n-butanol to the resulting solution above. The absorbance of the pink coloured extract was measured at 532 nm using spectrophotometer. The amount of MDA was calculated using a molar extinction coefficient of 1.56×10^5^M^-1^cm^-1^ and expressed as µmoles of MDA formed per gram of tissue as shown below.

MDA (µmoles/g tissue/ml) = absorbance/1.56×10^5^ *concentration of tissue in sample in (g/ml)

**Table 1:** Qualitative phytochemical screening result of crude methanol extract and solvent fractions of *Ocimum canum*

Samples	Saponins	Phenolics	Flavonoids	Tannins	Steroids	Carotenoids	Phlobtanins	Alkaloids	Triterpenes	Glycosides	Cardenolides
CROC	**++**	**++**	**++**	**+**	**-**	**++**	**-**	**+**	**++**	**+**	**-**
HXOC	**+**	**+**	**+**	**+**	**-**	**++**	**-**	**+**	**++**	**-**	**-**
ETOC	**++**	**+**	**+**	**+**	**-**	**+**	**-**	**+**	**+**	**+**	**-**
HMOC	**+**	**+**	**+**	**+**	**-**	**+**	**-**	**+**	**+**	**++**	**-**

OC: *Ocimum canum* CROC: Crude extract of OC, HXOC: Hexane fraction of OC, ETOC: ethylacetate fraction of OC, HMOC: aqueous-methanol fraction of OC

## Results

**Acute toxicity: **The LD50 of the crude extract was determined to be greater than 5000 mg/kgbw. This is considered non-toxic.

**Figure 1:**
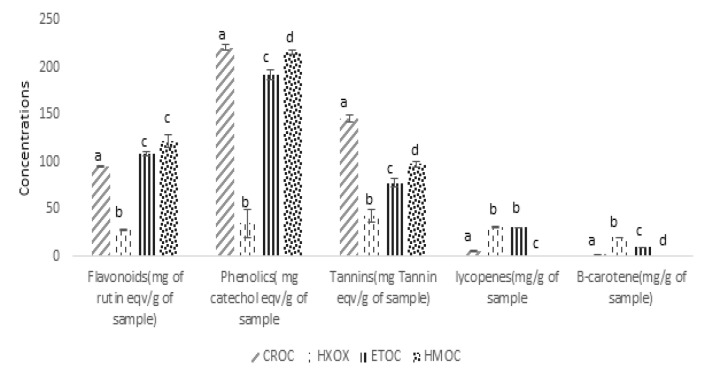
Quantitative phytochemical contents of crude methanol extract and solvent fractions of leaves Ocimum canum

**Phytochemical analysis: **The results of the qualitative and quantitative screening of the crude methanol extract and solvent fractions of *Ocimum canum *are presented in table 1 and figure 1, respectively. Table 1 showed that saponin, phenolic, flavonoids, tannins, carotenoids, triterpenes and alkaloids were present in all the samples (crude and fractions). Figure 1 showed quantitatively the flavonoids, phenolic, tannins, lycopene and β-carotene content of all the samples. A significantly (p<0.05) higher levels of flavonoids, phenolics and tannins were observed in the crude extract and the aqueous-methanol solvent fraction (HMOC). Conversely, the hexane and ethyl acetate solvent fractions revealed significantly (p<0.05) higher levels of carotenoids (lycopenes and β- carotenes).

OC: *Ocimum canum,* CROC: Crude extract of OC, HXOC:Hexane fraction of OC, ETOC: ethyl acetate fraction of OC, HMOC: aqueous-methanol fraction of OC

Values are mean ± SEM, n=3. Superscripts**:** Values (bars) bearing the same superscript are not statistically different (p>0.05).

***In vitro*** **antidiabetic activity: **The result of the α-amylase and α-glucosidase inhibitory activity of the crude methanol extract of *Ocimum canum* and its solvent fractions is presented in table 2. The *Ocimum canum *leave extract/fractions showed a poor inhibition of α-amylase and α-glucosidase activities relative to the standard drug Acarbose with IC_50_s greater than 2500µg/ml.

**Table 2:** Inhibitory effect (IC50) of crude methanol extract of Ocimum canum and its fractions on α-amylase and α-glucosidase activity *in vitro*

Sample	α-amylase IC_50_ (µg/ml)	α-glucosidase IC_50_ (µg/ml)
CROC	3135.5±60.0^c^	N.I
HXOC	2802.0±58.0^b^	N.I
ETOC	2945.8±137^b^	N.I
HMOC	6803.6±786.0^b^	4239.8±337.0^b^
ACAB	116.1±1.3^a^	482.05±7.4^a^

OC: *Ocimum canum* CROC: Crude extract of OC, HXOC:Hexane fraction of OC, ETOC: ethylacetate fraction of OC, HMOC: aqueous-methanol fraction of OC, ACAB: Acarbose, NI: No significant inhibition. Values are mean ± SEM, n=3 Superscripts**:** Values bearing the same superscript along a column are not statistically different (p>0.05).

**Figure 2:**
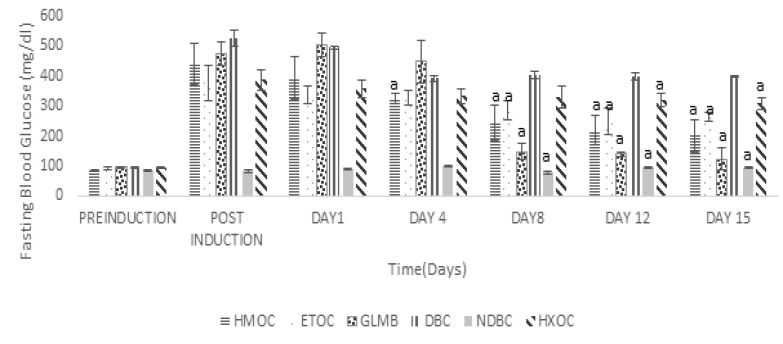
Effect of crude methanol extract of Ocimum canum on fasting blood glucose of diabetic wistar rats

**Figure 3:**
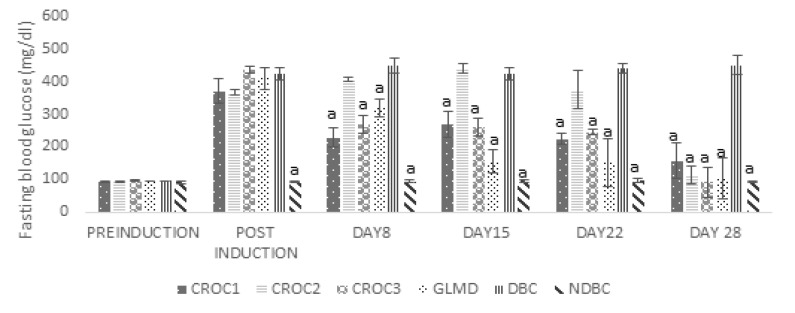
Effect of fractions of methanol extract of Ocimum canum on fasting blood glucose of diabetic Wistar rats

**Figure 4:**
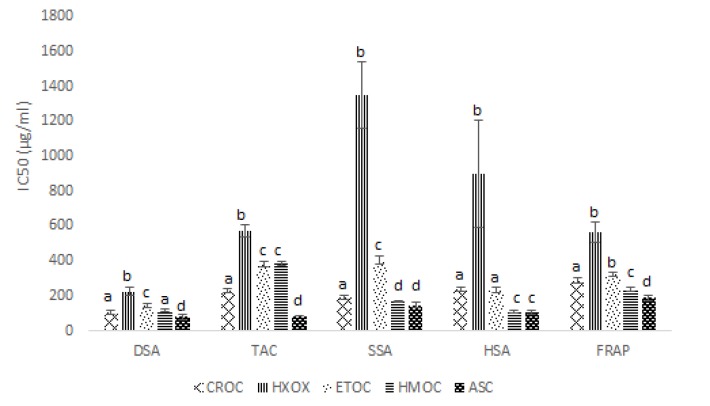
Histological sections of pancreas of the treated diabetic Wistar rats (H&E, mag x 400).

***In vivo*** **antidiabetic activity: **The result of the *in vivo* antidiabetic activity of the crude methanol extract and solvent fractions *Ocimum canum* leaves is presented in figure 2, figure 3 and table 3. The crude methanol extract (CROC) and the standard drug glibenclimide (GLMD) significantly (p<0.05) reduced the fasting blood glucose of the diabetic Wistar rats after 28 days in a dose-dependent manner compared to the untreated diabetic rat (figure 2), this was evident from the 8^th^ day of treatment. The effect of the crude extract / solvent fractions on serum insulin level and the change in fasting blood glucose (in percentage) are presented in table 3. The crude extract at a dose of 300 mg/kgbw (CROC3) and the standard drug, glibenclamide at 5 mg/kgbw significantly reduced the fasting blood glucose by 78.88% and 74.57% after 28 days respectively. They also caused a slight increase in insulin level relative to the diabetic control group. For the solvent fractions, the highest percentage reduction in fasting blood sugar relative to the diabetic control and the other solvent fractions was observed with the aqueous-methanol solvent fraction with a concomitant significant (p<0.05) increase in serum insulin in the treated groups. This was evident from the 4^th^ day of treatment. The histopathological sections of the pancreas of the diabetic Wistar rats with different treatments are presented in figure 4. The result showed that the normal control showed unremarkable pancreas, revealing evenly and densely distributed beta-cells. Some few scattered islet cells were observed in the groups treated with the aqueous-methanol and the ethyl acetate solvent fractions. The group treated with hexane solvent fraction and the diabetic control showed a highly distorted islet and acinar cells in their pancreas.

OC:*Ocimum canum, *CROC1: administered with crude methanol extract of OC (100 mg/kgbw)*,* CROC2: administered with crude methanol extract of OC (200 mg/kgbw)*,* CROC3: administered with crude methanol extract of OC (300 mg/kgbw), GLMD: administered with glibenclamide (5 mg/Kgbw), DBC: Diabetic control administered 10% DMSO, NDBC: Non-diabetic control administered 10% DMSO. Values are mean ± SEM, n=5. ^ a ^statistically different from diabetic untreated control group (p<0.05).

OC: *Ocimum canum,* ETOC: administered with ethylacetate fraction of OC (100 mg/kgbw), HMOC: administered with aqueous-methanol fraction of OC (100 mg/kgbw), HXOC: administered with hexan fraction of OC (100mg/kgbw), GLMD: administered with glibenclimide (5 mg/Kgbw), DBC: Diabetic control administered 10% DMSO, NDBC: Non-diabetic control administered 10% DMSO. Values are mean±SEM, n=5.

^ a ^statistically different from diabetic untreated control group (p<0.05).

**Table 3:** Effect of the crude methanol extract / solvent fractions of Ocimum canum on fasting blood glucose and serum insulin of diabetic wistar rats

Groups	After induction	After treatment	Serum insulin (µIU/ml)	Change in FBS (%)
CROC1	370.4±28.9	157.0±7.5^c^	12.77±0.26^d^	57.60
CROC2	366.4±7.2	114.5±8.1^b^	12.99±0.21^d^	68.75
CROC3	437.2±28.7	127.07±10.7^b^	12.95±0.37^d^	69.81
HMOC	419.0±69.6	200.5±12.9^d^	13.79±1.91^bcd^	52.60
ETOC	378.0±56.3	263.5±14.5^e^	13.24±1.02^bcd^	24.40
HXOC	387.0±34.7	308.0±20.1^f^	9.34±2.10^a^	20.41
GLMB	472.0±39.1	124.3±37.6^b^	14.87±2.90^b^	73.60
DBC	524.0±28.2	429.3±1.73^h^	9.20±0.24^a^	18.00
NDBC	83.2±2.7	90.0±6.70^a^	15.6±0.56^c^	----

*OC Ocimum canum, *CROC1: administered with crude methanol extract of OC (100 mg/kgbw) *,* CROC2: administered with crude methanol extract of OC (200 mg/kgbw) *,* CROC3: administered with crude methanol extract of OC (300 mg/kgbw) ETOC: administered with ethylacetate fraction of OC (100 mg/kgbw), HMOC: administered with residual aqiueous-methanol fraction of OC (100 mg/kgbw), HXOC: administered with hexane fraction of OC (100 mg/kgbw), GLMB: administered with glibenclimide (5 mg/Kgbw), DBC: Diabetic Control administered 10% DMSO, NDBC: Non-diabetic control administered 10% DMSO. FBS: Fasting blood glucose. Values are mean ± SEM, n=5. **Superscripts:** Values bearing the same superscript along a column are not statistically different (p>0.05).

OC: *Ocimum canum,* ETOC: administered with ethylacetate fraction of OC (100 mg/kgbw), HMOC: administered with aqueous-methanol fraction of OC (100 mg/kgbw), HXOC: administered with hexan fraction of OC (100 mg/kgbw), GLMD: administered with glibenclimide (5 mg/Kgbw), DBC: Diabetic control administered 10% DMSO, NDBC: Non-diabetic control administered 10% DMSO

**Figure 5:**
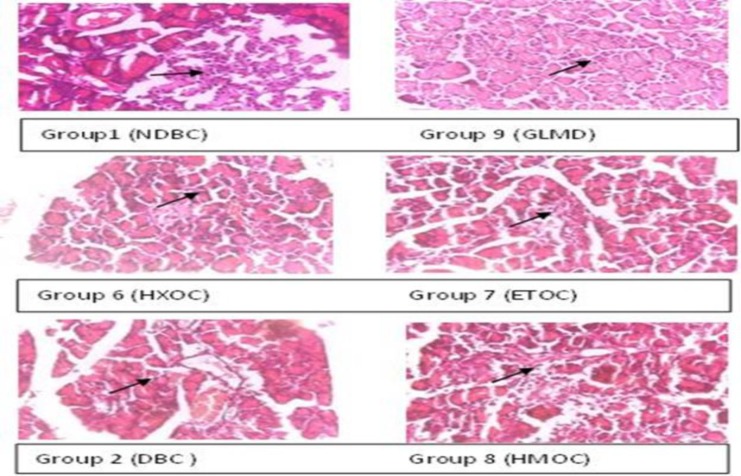
Antioxidant activity expressed in terms of IC50 (µg/ml) of Ocimum canum leaf exracts/fractions

***In vitro*** **antioxidant activity: **The *in vitro* antioxidant activity of the crude extract and its solvent fractions are presented in figure 5. The methanol crude extract and the solvent fractions showed a dose-dependent antioxidant and radical scavenging activity. The activities observed were in the following orders: ASC>CROC>HMOC>ETOC>HXOC for Superoxide scavenging assay (SSA); ASC>HMOC>ETOC>CROC>HXOC, for hydroxyl scavenging assay (HSA); ASC>CROC>HMOC> ETOC>HXOC for DPPH scavenging assay (DSA); ASC>HMOC>CROC>ETOC>HXOC for ferric reducing power (FRAP); and ASC>CROC>ETOC>HMOC>HXOC for total antioxidant activity (TAC).

OC: *Ocimum canum,* ETOC: ethylacetate fraction of OC, HMOC: aqiueous-methanol fraction of OC, HXOC: hexane fraction of OC, ASC: Ascorbic acid. Values are mean ± SEM, n=3. **Superscripts:** Values bearing the same superscript along a column are not statistically different (p>0.05).

**Figure 6:**
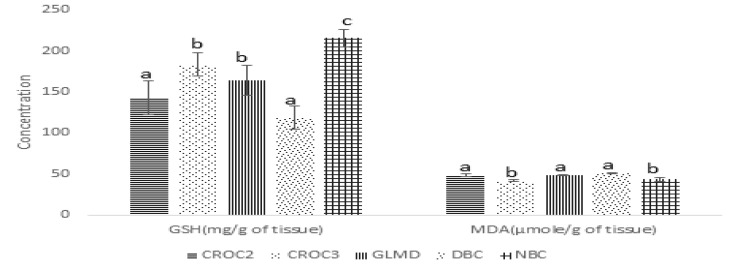
Effect of crude methanol extract of Ocimum canum leaves on oxidative stress markers in diabetic Wistar rats

***In vivo*** **antioxidant activity: **The effect of the crude extract on oxidative stress markers is presented in figure 6 and table 4. The result showed that the diabetic control had a significantly (p<0.05) lower GSH (reduced glutathione) and higher MDA (malonaldehyde) level while the treated groups (CROC2, CROC3, GLMD) and normal control group (NDBC) had relatively higher GSH and lower MDA. Table 4 presents the result of the effect of the solvent fractions of the methanol extract of *Ocimum canum *respectively on antioxidant status of diabetic wistar rats and non-diabetic wistar rats. The result showed that superoxide dismutase (SOD) and glutathione peroxidase (GSH-Px) increased in diabetic control group (DBC) relative to the normal control (NDBC). The MDA level in DBC group was higher relative to other groups. The GSH level and catalase activity were significantly (p<0.05) lower in the diabetic control than in the normal control. The groups treated with the aqueous-methanol solvent fraction had significantly lower SOD and GSH-Px when compared to the diabetic control group, but was lower than that observed in the normal control group. The GSH and catalase activity also showed similar trend - higher than what was observed in diabetic control but lower in the normal control.

OC: *Ocimum canum,* CROC2: administered with crude methanol extract of OC (200mg/kgbw)*,* CROC3: administered with crude methanol extract of OC (300 mg/kgbw), GLMD: administered with glibenclamide (5 mg/Kgbw), DBC: Diabetic control administered 10% DMSO, NDBC: Non-diabetic control administered 10% DMSO. Values are mean±SEM, n=5. Superscripts: Values bearing the same superscript along a column are not statistically different (p>0.05).

**Table 4:** Effect of fractions of methanol extract of Ocimum canum on oxidative stress markers in rats

**Groups**	**GSH (mg/g of tissue)**	**MDA(µmole/g of tissue)**	**Catalase (U/g of tissue)**	**SOD (U/mg of tissue)**	**GSHPx(nmoleGSH/min/mg of tissue)**
HMOC	193.6±3.9^b^	76.22±4.98^a^	9.26±1.60^b^	0.142±0.02^c^	0.297±0.020^a^
ETOC	184.6±10.6^a^	82.59±4.76^a^	9.68±1.40^b^	0.169±0.04^b^	0.313±0.020^b^
HXOC	179.4±11.3^a^	88.40±7.3^a^	9.69±3.20^b^	0.181±0.06^b^	0.312±0.018^b^
GLMD	186.8±6.9^a^	80.36±6.50^a^	9.23±0.63^b^	0.165±0.02^b^	0.308±0.005^b^
DBC	171.2±6.8^a^	96.63±6.10^a^	7.5±1.90^a^	0.220±0.04^a^	0.329±0.005^b^
NDBC	232.5±9.7^c^	57.72±1.39^b^	12.8±1.90^c^	0.120±0.01^c^	0.284±0.010^a^

OC: *Ocimum canum,* ETOC: administered with ethylacetate fraction of OC (100 mg/kgbw), HMOC: administered with aqueous-methanol fraction of OC (100 mg/kgbw), HXOC: administered with hexan fraction of OC (100 mg/kgbw), GLMD: administered with glibenclimide (5 mg/Kgbw), DBC: Diabetic Control administered 10% DMSO, NDBC: Non-Diabetic control administered 10% DMSO. Values are mean±SEM, n=5. **Superscripts:** Values bearing the same superscript along a column are not statistically different (p>0.05).

## Discussion

The use of ethno botanicals has a long folkloric history for the treatment of diabetic conditions as well as oxidative stress-related conditions (1, 15); however, it still suffers from extensive scientific validation. Therefore, the search for more effective and safer antidiabetic/ hypoglycemic/ antioxidant agents remain an important area of research. In this present study, the leaves of *Ocimum canum* were evaluated for antidiabetic and antioxidant activity, owing to its use in ethnomedicine locally in southeastern part of Nigeria. The phytochemical analysis of crude extracts of *Ocinum* *canum* leaves revealed the presence of several phytochemicals: saponins, phenolics, flavonoids, tannins, carotenoids, alkaloids, triterpenes, and glycosides. The solvent fractions all had saponins, phenolics, flavonoids, tannins, carotenoids and alkaloids in common but in varying compositions. Steroids were found in the ethyl acetate fractions while lycopenes and β-carotenes were significantly higher in ethyl acetate and hexane fractions. This could be attributed to the solubility of these phytochemicals in solvents of different polarity. According to Martson and Hostettman (23), polarity is an important factor that determines the solubility of compounds. Less polar flavonoids are more soluble in ethyl acetate, chloroform, diethyl ether while the more polar flavonoids are largely extracted with a mixture of water and alcohol. This explains the high flavonoid, tannin and phenolic contents in the aqueous-methanol fractions. Carotenoids are hydrophobic owing to their long hydrocarbon chains and as such are extractable by non-polar organic solvents (24). 

This finding is in line with the works of Iloki-Assanga *et al.* (25). Hexane fractions of *Buceras L.* and *Phoradendron calofornicum* revealed presence of carotenes, triterpenes/steroids, lactonic acid while the aqueous and ethanol fractions revealed the presence of diverse range of compounds including saponins, phenols, tannins, amines, amino acids and flavonoids/anthocyanins. α-amylase and α-glucosidase are carbohydrate digesting enzymes. They are required to hydrolyze complex polysaccharides and disaccharides respectively to release glucose into the general circulation. Inhibition of these enzymes has proven to be an efficient approach in controlling postprandial sugar (26). In this present study, The *Ocimum canum* crude extract and solvent fractions showed a poor inhibitory activity against the two enzymes relative to Acarbose, a standard drug that exerts its activity by inhibiting the aforementioned digestive enzymes. *Ocimum* genera have been reported widely to have antidiabetic potentials. *Ocimum basilicum *(27, 28), *Ocimum tenuiflorum* (29), *Ocimum sanctum *(28) have all been reported to exhibit strong inhibition against α-amylase and α-glucosidase. However, this present study, *Ocimum canum* did not record any significant inhibitory activity against the two enzymes. This finding may be attributed to specie differences. Phytochemical composition is expected to vary to a degree between species within a genus (30). Streptozotocin has been shown to cause direct irreversible damage (necrosis) to β-cells of pancreatic islets of Langerhans, resulting in degranulation and consequent loss of insulin secretions. Clarification of the regenerating potential in experimentally-induced diabetic animal is of huge interest in finding alternative therapy for diabetes. Plant extracts with high polyphenolic contents have been reported to potentiate insulin secretion from residual and regenerated β-cells or its action of releasing bound insulin from β-cells, by inhibiting ATP sensitive K^+^ channels like Glibenclimide (31). In this present study, the crude extract and its aqueous-methanol fraction significantly reduced the fasting blood sugar by 69.81% and 52.60%, respectively, and concomitantly increased the serum insulin levels in the treated diabetic Wistar rats. This activity may be linked to some class of compounds-phenolics, tannins and flavonoids- which were higher in the crude extract and aqueous-methanol solvent fraction. Histological sections of the pancreas showed that the normal control (NDBC) group and the group treated with standard drug, glibenclimide (GLMD) group revealed a large number of β-cells evenly distributed in the islets of Langerhans. In contrast, small-volumed and irregular shaped islets were observed in aqueous methanol (HMOC), and ethyl acetate fraction (ETOC) as against the diabetic control, DBC showed highly disordered islets and no visible β-cells. This observation suggests that this extract may contain compounds that protect the beta-cells from further oxidative damages, potentiate the secretion of insulin from the residual beta cells and consequently may activate the uptake of glucose by peripheral tissues such as muscle. 

Oxidative stress is a suggested mechanism for the induction and progression of diabetes and diabetic complications, which results from imbalance between prooxidants / radical generating and antioxidants/radical scavenging system (26)*.* Hyperglycemia is strongly associated with increased superoxide generation via the mitochondrial system (32-34). Although it is a weak oxidant, it gives rise to the generation of powerful and dangerous hydroxyl radicals as well as singlet oxygen, both of which contribute to oxidative stress. The hydroxyl radical is regarded as a detrimental reactive oxygen species in pathophysiological processes and capable of damaging almost every biomolecule. It is also implicated in carcinogenesis, mutagenesis, cytotoxicity and pathogenesis of chronic disease (35). The antioxidant activity of the extracts *in vitro* was evaluated by DPPH, O_2_^*^, HO^*^ scavenging and reducing power assays. The result of this study revealed that the methanolic extract of *Ocimum canum *possesses phytochemicals with high capacity to scavenge free radicals and reduce highly damaging oxidative intermediates. These groups of phytochemicals may be largely more soluble in aqueous-methanol as shown by the quantitative phytochemical content and the median inhibitory concentrations (IC_50_s) for the different *in vitro* antioxidant assays of the aqueous methanol fraction of *Ocimum canum *relative to the other solvent fractions. These components could be flavonoids and phenolics which have been shown to be highly antioxidative via their reducing power or electron/hydrogen donating/transfer potentials (36)*. * This finding is consistent with that reported by (37-39). Antioxidant enzymes as well as non-enzymatic antioxidants are the first line of defense against reactive oxygen species (ROS) induced oxidative damages in a living organism. Superoxide dismutase (SOD), glutathione peroxidase (GPx), and catalase (CAT) are the three major antioxidant enzymes that remove free radicals *in vivo*. SOD protects tissues against reactive oxygen species by dismutating superoxide into molecular oxygen and hydrogen peroxide which are also capable of damaging the cell membrane and other biological structures. CAT on the other hand is responsible for the detoxification of significant amounts of hydrogen peroxide (H_2_O_2_) (23). GPx plays a central role in the catabolism of H_2_O_2_ and detoxification of endogenous metabolic peroxides and hydroperoxides using reduced glutathione (GSH) (26). GSH functions as a free radical scavenger and is an essential co-substrate for GSH-Px (26). Lipid peroxidation is one of the characteristic features of chronic diabetes. The increased free radicals produced usually react with polyunsaturated fatty acids in cell membranes, leading to lipid peroxidation, which in turn results in elevated production of free radicals (26). In this present study, malonaldehyde, SOD and GSH-Px activity increased significantly while GSH and CAT activity decreased in the diabetic control group compared to the normal control. Treatment with the crude methanol extract of *Ocimum canum* and its aqueous-methanol fraction significantly reversed these trends of antioxidant status in the diabetic rats to levels that compared with the normal control group. A significant difference was not observed between the glibenclimide treated and the diabetic control group. An indication that glibenclamide may not posse significant antioxidant activity. All the improved outcomes recorded with treatment of diabetes using glibenclimide might be attributable to its ability to ameliorate hyperglycemic condition which initiates and potentiates the oxidative stress in diabetic condition. The increased MDA and decreased level of GSH are indicative of oxidative stress in the untreated diabetic groups. The increased SOD and GSH-Px activity in the diabetic control group may be a compensatory mechanism to counter the high level oxidative stressors like superoxide, hydroxyl radicals and hydrogen peroxides which are common free radicals associated with hyperglycemic conditions. The extract/fraction which reversed this trend probably acted by assisting in mopping up some of the free radicals and reducing the strain on the antioxidant system. The decreased level of catalase in the untreated diabetic rats might be as a result of inhibition of the enzyme by high level of superoxide or glycation of the hemprotein as a result of hyperglycemia (40). 

Kono and Fridovich in 1982 demonstrated that superoxide specifically inhibits catalase activity using an enzymatic source of superoxide (41). This observation is the basis for the idea that SOD and catalase may be mutually protective set of enzymes. Hyperglycemia in diabetes leads to an overdrive of the electron transport chain in the mitochondria, which consequently results in the generation of excess superoxide radicals (40). The elevated blood glucose in the diabetic control rats could have enhanced superoxide radical generation which consequently inhibited the catalase activity. The high SOD and GSH-Px activity recorded in the diabetic untreated rats might be a consequence of increased superoxide and hydrogen peroxide generated by enhanced SOD as observed by Kharazi-Nejad *et al.* (42). There are conflicting results with regard to the GSH-Px CAT and SOD activity in diabetic condition in previous studies. Some studies reported reduced activities while some reported increased activity (26, 31, 40, 43). Kharazi-Nejad *et al. *(42) and Ramanathan *et al. *(44) reported increases in SOD and CAT following STZ-induction of diabetes. Conversely, Anwar and Meki (42) reported a decline in the activity of these enzymes following a diabetic untreated condition. These differences in result may be accounted for by the following; degree of oxidative stress, nature of the stressors or inducing agents, mechanisms of oxidative assault and duration of assault condition or treatment. The inducing agents, inducing doses and treatment durations for some of the reported studies varied.

In conclusion, the findings of this study show that the methanolic extract of *Ocimum canum *has high potential to ameliorate hyperglycemic condition as well as the associated oxidative stress. The aqueous-methanol solvent fraction exhibited the highest activity. This provides scientific evidence for the ethno medicinal use of this plant and a baseline data for further work to characterize the extract.
